# Utilization of Subcutaneous Cardioverter-Defibrillator in Poland and Europe–Comparison of the Results of Multi-Center Registries

**DOI:** 10.3390/ijerph18137178

**Published:** 2021-07-05

**Authors:** Maciej Kempa, Andrzej Przybylski, Szymon Budrejko, Tomasz Fabiszak, Michał Lewandowski, Krzysztof Kaczmarek, Mateusz Tajstra, Marcin Grabowski, Przemysław Mitkowski, Stanisław Tubek, Ewa Jędrzejczyk-Patej, Radosław Lenarczyk, Dariusz Jagielski, Janusz Romanek, Anna Rydlewska, Zbigniew Orski, Joanna Zakrzewska-Koperska, Artur Filipecki, Marcin Janowski, Tatjana Potpara, Serge Boveda

**Affiliations:** 1 Department of Cardiology and Electrotherapy, Medical University of Gdansk, 80-210 Gdansk, Poland; kempa@gumed.edu.pl; 2 Medical College, University of Rzeszow, 35-959 Rzeszow, Poland; a_przybylski-65@wp.pl (A.P.); januszromanek@wp.pl (J.R.); 3 Cardiology Department with the Acute Coronary Syndromes Subdivision, Clinical Provincial Hospital No. 2, 35-301 Rzeszow, Poland; 4 Department of Cardiology and Internal Diseases, Collegium Medicum, Nicolaus Copernicus University, 85-094 Bydgoszcz, Poland; tfabiszak@wp.pl; 5 2nd Department of Arrhythmia, National Institute of Cardiology, 04-628 Warsaw, Poland; mlewandowski@ikard.pl; 6 Department of Electrocardiology, Medical University of Lodz, 90-647 Lodz, Poland; krzysztof.kaczmarek@umed.lodz.pl; 7 3rd Department of Cardiology, Silesian Centre for Heart Diseases, School of Medicine with the Division of Dentistry in Zabrze, Medical University of Silesia, 40-055 Katowice, Poland; mateusztajstra@wp.pl; 8 1st Chair and Department of Cardiology, Medical University of Warsaw, 02-091 Warsaw, Poland; marcin.grabowski@wum.edu.pl; 9 1st Department of Cardiology, Chair of Cardiology, Karol Marcinkowski University of Medical Sciences, 61-701 Poznan, Poland; przemyslaw.mitkowski@ump.edu.pl; 10 Department of Heart Diseases, Wroclaw Medical University, 50-367 Wroclaw, Poland; stanislaw.tubek@gmail.com; 11 Department of Cardiology, Congenital Heart Diseases and Electrotherapy, Medical University of Silesia Silesian Centre for Heart Diseases, 41-800 Zabrze, Poland; ewajczyk@op.pl (E.J.-P.); radle@poczta.onet.pl (R.L.); 12 Department of Cardiology, Centre for Heart Diseases, 4th Military Hospital, 50-981 Wroclaw, Poland; dariuszjagielski@gmail.com; 13 Institute of Cardiology, Faculty of Medicine, Jagiellonian University Medical College, 31-008 Kraków, Poland; annarydlewska@op.pl; 14 Department of Electrocardiology, The John Paul II Hospital, 31-202 Krakow, Poland; 15 Department of Cardiology and Internal Diseases, Military Institute of Medicine, 04-141 Warsaw, Poland; zorski@wim.mil.pl; 16 1st Department of Arrhythmia, National Institute of Cardiology, 04-628 Warsaw, Poland; jzakrzewska@ikard.pl; 17 1st Department of Cardiology, School of Medicine in Katowice, Medical University of Silesia, 40-055 Katowice, Poland; arturfilipecki@wp.pl; 18 Chair and Department of Cardiology, Medical University of Lublin, 20-059 Lublin, Poland; marcin.janowski@umlub.pl; 19 School of Medicine, Belgrade University, 11000 Belgrade, Serbia; tanjapotpara@gmail.com; 20 Cardiology, Cardiac Arrhythmias Management Department, Clinique Pasteur, 31076 Toulouse, France; sboveda@clinique-pasteur.com

**Keywords:** sudden cardiac death, ventricular arrhythmia, implantable cardioverter-defibrillator, subcutaneous implantable cardioverter-defibrillator

## Abstract

The implantation of a subcutaneous cardioverter-defibrillator (S-ICD) may be used instead of a traditional transvenous system to prevent sudden cardiac death. Our aim was to compare the characteristics of S-ICD patients from the multi-center registry of S-ICD implantations in Poland with the published results of the European Snapshot Survey on S-ICD Implantation (ESSS-SICDI). We compared data of 137 Polish S-ICD patients with 68 patients from the ESSS-SICDI registry. The groups did not differ significantly in terms of sex, prevalence of ischemic cardiomyopathy, concomitant diseases, and the rate of primary prevention indication. Polish patients had more advanced heart failure (New York Heart Association (NYHA) class III: 11.7% vs. 2.9%, NYHA II: 48.9% vs. 29.4%, NYHA I: 39.4% vs. 67.7%, *p* < 0.05 each). Young age (75.9% vs. 50%, *p* < 0.05) and no vascular access (7.3% vs. 0%, *p* < 0.05) were more often indications for S-ICD. The percentage of patients after transvenous system removal due to infections was significantly higher in the Polish group (11% vs. 1.5%, *p* < 0.05). In the European population, S-ICD was more frequently chosen because of patients’ active lifestyle and patients’ preference (both 10.3% vs. 0%, *p* < 0.05). Our analysis shows that in Poland, compared to other European countries, subcutaneous cardioverters-defibrillators are being implanted in patients at a more advanced stage of chronic heart failure. The most frequent reason for choosing a subcutaneous system instead of a transvenous ICD is the young age of a patient.

## 1. Introduction

The implantation of a subcutaneous cardioverter-defibrillator (S-ICD) has been increasingly used to treat patients at risk of sudden cardiac death due to ventricular arrhythmias. Such a solution may in many cases replace a traditional cardioverter-defibrillator system with transvenous leads (transvenous cardioverter-defibrillator, TV-ICD) [1,2]. According to the European and American guidelines, S-ICD is contraindicated if a patient requires permanent cardiac pacing, including resynchronization therapy, or has a history of ventricular tachycardia that is possibly eligible for termination with antitachycardia pacing [3,4]. S-ICD systems have been used in Poland since 2014 [5,6]. Due to complex rules of reimbursement, the number of procedures was initially low. Not until the beginning of 2019 has S-ICD implantation been cleared for a full refund by the National Health Fund [7]. To our knowledge, there is no data that compare Poland with other European countries in terms of the population characteristics of S-ICD patients and the indications for the selection of that particular method of treatment.

The aim of our study was to compare patients’ populations and indications for S-ICD implantation in Poland with available European data.

## 2. Materials and Methods

The analysis incorporates data gathered between May 2020 and March 2021 in the multi-center registry of S-ICD implantations in Poland, run by the Heart Rhythm Section of the Polish Cardiac Society without any support from the industry. Participation in the registry did not in any way influence the qualification of patients for the procedure, procedural technique, or follow-up care in any of the centers involved. Data was collected and entered into the registry database when the hospitalization of a patient was finished. Collected information included: age, gender, underlying disease, data regarding indications for S-ICD implantation, basic electrocardiographic measurements, procedural technique, and postoperative course, with any possible complications during hospitalization. Data were compared with the published results of the European Snapshot Survey on S-ICD Implantation (ESSS-SICDI) [8], which collected data from 20 European centers (eight from France, six from Poland, two from Germany and Italy, and one from Austria and Switzerland). Altogether, 429 patients were reported during the period from April to June 2017, and for 383 of them information regarding the type of implanted device was available. In 76, S-ICD was implanted, and in the remaining 307 patients, TV-ICD was implanted. For the purpose of our analysis we only selected data from those 76 European patients with S-ICD but then excluded eight patients reported by Polish centers. Thus, finally, a group of 68 S-ICD patients from the ESSS-SICDI survey was taken for the comparison [9].

Continuous variables were presented as the mean and standard deviation or median and interquartile range, in the case of a non-normal distribution. Categorical parameters were presented as numbers and percentages. The normality of distribution was tested with the Shapiro–Wilk test. The χ^2^ test, and the Student t-test or Mann–Whitney U test (depending on the analysis of distribution and variance) were used to compare the groups, as appropriate for a given variable. A *p* value <0.05 was considered statistically significant. The statistical analysis was performed with the use of Statistica 13.1 software (TIBCO Software Inc., Palo Alto, CA 94304, USA).

## 3. Results

During the period from May 2020 to March 2021, data from 147 patients have been reported to the registry of S-ICD implantations in Poland, which corresponds to 90% of all S-ICD implantations performed in our country during that time. In that population, 137 patients had undergone primary implantation, and the remaining 10 had undergone a device exchange due to an elective replacement indicator (those 10 patients were excluded from further analysis). Altogether, 15 centers reported on 34 female and 103 male patients (age 15 to 79, mean ± SD 43.4 ± 15.3), the majority remaining in the NYHA II or NYHA I functional class, 67 (48.9%) and 54 patients (39.4%), respectively. The left ventricle ejection fraction (LVEF) in this group ranged between 10 and 80%, (median [IQR] 33 (25–57)). In 91 cases (66.4%), the S-ICD system was implanted for the primary prevention of sudden cardiac death (SCD). Nonischemic cardiomyopathy was the most prevalent underlying disease, with 64 patients (46.7%). The main reason for choosing S-ICD rather than TV-ICD was the young age and long life expectancy of a patient (75.9%). Among patients with an LVEF below 35%, only two patients had a QRS complex width of over 150 ms (nonleft bundle branch block morphology) but were not considered for transvenous cardiac resynchronization therapy because of a high risk of infective complications (one patient had a chronic infection and a history of extraction of a transvenous cardioverter-defibrillator, and the other one had a history of infective endocarditis). Detailed data are shown in Table 1 and Table 2.

Our data were compared with published results from the ESSS-SICDI survey, which after exclusion of Polish S-ICD patients (*n* = 8), comprised 68 S-ICD patients (22 female), mostly remaining in the I or II functional NYHA class (46 [67.7%] and 20 [29.4%], respectively). In 24 patients (35.5%), coronary artery disease was the underlying disease, while no structural heart disease was reported in another 20 (29.4%). The LVEF interquartile range was between 25% and 60% (median 50%). In most cases (63.2%), S-ICD was implanted for the primary prevention of SCD. S-ICD (and not TV-ICD) was chosen mostly due to the young age of patients—this reason prevailed in 34 cases (50%). The clinical data from the two groups of patients are presented in Table 2.

The comparative analysis showed similar demographic data: the prevalence of ischemic cardiomyopathy and concomitant diseases (except for a higher prevalence of COPD in patients from ESSS-SICDI) in the two analyzed groups. European patients were more often (*p* < 0.05) in NYHA class I (67.7% vs. 39.4%) but less often in NYHA class II and class III (29.4% vs. 48.9% and 2.9% vs. 11.7%, respectively, both *p* < 0.05). The median LVEF in the Polish population was numerically lower (38.3% vs. 50%), but statistical significance could not be determined due to the lack of source data and data distribution from the ESSS-SICDI population. Left bundle branch block was more frequent in the European population (4.4% vs. 0%). Furthermore, in the European group S-ICD was slightly more frequently implanted in patients without structural heart disease, but that comparison did not reach statistical significance (29.4% vs. 18.3%).

Reasons to implant S-ICD and not TV-ICD showed significant differences between the groups. In the Polish group, the young age of patients (75.9% vs. 50%, *p* < 0.001) and no vascular access (7.3% vs. 0%, *p* < 0.05) were significantly more often represented. Additionally, the percentage of patients with a history of transvenous system removal due to infectious complications was significantly (*p* < 0.05) higher in the Polish group (11% vs. 1.5%). On the contrary, in the European population S-ICD was more frequently chosen because of patients’ active lifestyle (10.3%) and patients’ preference (10.3%), whereas in our group no center declared such reasons (both *p* < 0.001). Anticipated lead-related complications in the case of transvenous implantation were also reported more often in the European group (26.5% vs. 0%). A detailed comparison may be found in Table 3.

## 4. Discussion

S-ICD systems have been implanted in Poland since 2014, yet full reimbursement was introduced five years later. In addition, the sources of further limitations regarding the selection (and reimbursement) of S-ICD and not TV-ICD are still the payer (additional clinical requirements for the reimbursement) and the centers themselves (operators/center experience). At the same time, there are no reports on how such a situation with limited access to technology might influence factors that determine device choice and the clinical characteristics of the population that receives S-ICD. The multi-center registry run by the Heart Rhythm Section of the Polish Cardiac Society established a possibility for comparisons of Polish S-ICD patients with other groups; it also allowed one to compare the indications and reasons behind a choice for a specific device. The results of the 2017 European Snapshot Survey on S-ICD Implantation gave insight into the practices regarding S-ICD implantation in European countries. A comparison of data from both registries reveals some differences between Poland and the rest of Europe in terms of the clinical characteristics of patients undergoing S-ICD implantation and the indications for the procedure. As the source data of the ESSS-SICDI are not publicly open, our comparison is based on the results published by Jędrzejczyk–Patej et al. in 2018, analyzing a subpopulation of the patients from ESSS-SICDI with the exclusion of patients reported to that registry by Polish centers.

The main difference shown by our comparison of the two analyzed groups is the severity of heart failure at the time of S-ICD implantation. In the ESSS-SICDI group, as many as 67.7% of patients were in the NYHA I functional class, whereas in the Polish population it was only 39.4%. Opposite ratios were found for the NYHA III class because only 2.9% of European patients were reported to be in that class, compared to 11.7% in the Polish group. Additionally, the mean left ventricle ejection fraction was numerically lower in the Polish group. The above findings are concordant with those reported by Jędrzejczyk–Patej et al. in 2018; however, their older analysis was based on only eight S-ICD patients from Poland, implanted before the new reimbursement rules were set by the National Health Fund. Nonetheless, the previously observed trends that Polish patients at the time of implantation had more severe heart failure and lower LVEF seem to still be valid. That phenomenon may stem from three reasons. First, as shown by the results of the European Heart Failure Pilot Survey, Polish patients treated for chronic heart failure on an out-patient basis, as well as those referred to hospitals due to exacerbation of symptoms, had a lower LVEF and higher NYHA class than corresponding patients in other European countries. They less frequently already had an implantable cardioverter-defibrillator in place [10]. Consequently, at the time of implantation, their heart failure was already more advanced. The second reason is that in other European countries the decision to choose S-ICD may be based on such factors as patient’s preference, active lifestyle, and even cosmetic aspects (e.g., scar location after implantation) [8]. Due to that fact, S-ICD is being more often implanted in younger, physically active patients, who still remain in good condition and have less advanced heart failure. Reimbursement regulations in Poland do not consider such factors at all when it comes to decision-making on S-ICD implantation. Lastly, S-ICD in other European countries is more frequently implanted in patients without structural heart disease (and, consequently, with a better NYHA class and higher LVEF).

Interesting conclusions may be derived from the analysis of the reasons behind choosing S-ICD instead of TV-ICD. In Polish centers, a younger age was the most frequent reason to select S-ICD, and it was reported in 75.9% of patients. In other European centers, that factor was decisive in half of the patients. There was also a significant difference in the rate of patients with a history of removal of a previous transvenous system due to infections. In Poland, that was the case in over 11% of patients, whereas in the ESSS-SICDI population, it was only the case in one patient (1.5%). Together, active lifestyle and patient’s preference were the main reason for S-ICD implantation in over 20% of patients in the European population. In the Polish group such a reason was not reported at all. That discrepancy may be due to the lack of a strict definition of “young age” in both registries, which may bias the rate of that particular indication in either direction. One has to keep in mind, however, that the factor described as “active lifestyle” could have been underreported in the Polish population because cardiologists qualifying for S-ICD implantation who reported “young age” might also have understood other possible meanings under that category that were not specifically suggested, such as “active lifestyle”. However, the most important factors possibly influencing the reporting rate of specific indications are the reimbursement regulations in Poland. Current regulations do not list active lifestyle or patient’s preference among possible reasons justifying the choice of S-ICD for patients with a general indication for an ICD. That may explain why none of the participating centers in Poland reported such a factor, despite having the possibility to add any additional reasons. We feel obliged to comment also on the high rate of indication described as “anticipated lead-related TV-ICD complications” in the European group. Among Polish patients, no case of such an indication was reported. This may be due to the fact that in our understanding a history of lead-related complications clearly increases the risk of a future repeated occurrence. Therefore, records of patients with the indication “previous LR complications” were not additionally supplemented with another indication of an anticipated risk of repeated lead-related complications.

## 5. Limitations of the Study

The main limitation of the study is the time difference between data reporting to both registries. The results from the ESSS-SICDI were published in 2018, whereas data from Polish centers were collected until March 2021 and were more up to date. Nonetheless, the introduction of S-ICD in Poland was delayed by several years in relation to other European countries, and therefore the time of experience with the method in a given country from its start to registry data reporting may be similar [11]. The length of follow-up in our registry, as well as the character of data reported so far, did not allow for more extended comparisons of long-term clinical outcomes. Reliability of data in both registries may be another limitation. The registry of the Polish Heart Rhythm Section is being carried on by specific centers, with coordinators prompted to report data, and the appropriate contract is set by the agreement between the specific center and the Heart Rhythm Section. The ESSS-SICDI survey was performed on the basis of a voluntary participation of implanting centers and the use of completely anonymous data reporting. For the purpose of our comparison, relevant data were extracted from the available publication of the results of that registry (a bibliographic cohort).

In most countries, implantation of S-ICD is associated with a significantly higher cost compared to transvenous systems. Therefore, the financial issues and reimbursement regulations might have affected clinical decisions and biased the data.

What is of further note, the Polish registry contains data collected and reported in part during the COVID-19 pandemic. That might have changed and biased decisions in terms of device selection [12].

## 6. Conclusions

Our analysis shows that in Poland, when compared to other European countries, subcutaneous cardioverters-defibrillators are being implanted in patients at a more advanced stage of chronic heart failure. The most frequent reason for choosing subcutaneous-ICD rather than TV-ICD is the young age of a patient.

## Figures and Tables

**Table 1:** Clinical characteristics of patients in the study group.

Age [Years]; Mean (SD)	43.4 (15.3)
Male; *n* (%)	103 (75.2)
Sinus rhythm; *n* (%)	128 (93.4)
Underlying disease	
NICM; *n* (%)	64 (46.7)
ICM; *n* (%)	38 (27.7)
HCM; *n* (%)	6 (4.4)
LQTS; *n* (%)	5 (3.6)
BrS; *n* (%)	3 (2.2)
SQTS; *n* (%)	2 (1.5)
Myocarditis; *n* (%)	2 (1.5)
LVNC; *n* (%)	1 (0.7)
CPVT; *n* (%)	1 (0.7)
ToF; *n* (%)	1 (0.7)
Primary VF; *n* (%)	14 (10.2)
LVEF; median % (IQR)	33 (25–57)

NICM—nonischemic cardiomyopathy; ICM—ischemic cardiomyopathy; HCM—hypertrophic cardiomyopathy; LQTS—long QT syndrome; BrS—Brugada syndrome; SQTS—short QT syndrome; LVNC—left ventricular noncompaction; CPVT—catecholaminergic polymorphic ventricular tachycardia; ToF—tetralogy of Fallot; VF—ventricular fibrillation; LVEF—left ventricle ejection fraction, IQR—interquartile range.

**Table 2:** Comparison of clinical characteristics between the study group and the ESSS-SICDI group—Jędrzejczyk–Patej et al. [9].

	Europe	Poland	*p*
Total number of patients; *n* (%)	68 (100)	137 (100)	-
Age <18 years; *n* (%)	2 (2.9)	3 (2.2)	0.7426
Age >75 years; *n* (%)	0 (0)	0 (0)	-
Women; *n* (%)	22 (32.4)	34 (24.8)	0.2543
NYHA I; *n* (%)	46 (67.7)	54 (39.4)	0.0001
NYHA II; *n* (%)	20 (29.4)	67 (48.9)	0.0078
NYHA III; *n* (%)	2 (2.9)	16 (11.7)	0.0374
NYHA IV; *n* (%)	0 (0)	0 (0)	-
Ischemic etiology of HF; *n* (%)	24 (35.5)	38 (27.7)	0.2674
No structural heart disease; *n* (%)	20 (29.4)	25 (18.3)	0.0691
Primary prevention of SCD; *n* (%)	43 (63.2)	91 (66.4)	0.6515
Diabetes mellitus; *n* (%)	9 (13.2)	18 (13.1)	0.9846
Chronic kidney disease; *n* (%)	4 (5.9)	16 (11.7)	0.1879
COPD; *n* (%)	3 (4.4)	0 (0)	0.0133
AF/AFL; *n* (%)	4 (5.9)	9 (6.6)	0.8493
Sick sinus syndrome at implantation; *n* (%)	0 (0)	1 (0.7)	0.48
High degree AV block at implantation; *n* (%)	0 (0)	0 (0)	-
LVEF (%); median (IQR)	50 (25–60)	33 (25–57)	- *
Left bundle branch block; *n* (%)	3 (4.4)	0 (0)	0.0133
QRS 120–150 ms; *n* (%)	13 (19.1)	23 (16.8)	0.6798
QRS > 150 ms; *n* (%)	0 (0)	2 (1.5)	0.3167

HF—heart failure; SCD—sudden cardiac death; COPD—chronic obstructive pulmonary disease; AF—atrial fibrillation; AFL—atrial flutter; AV—atrio-ventricular; LVEF—left ventricle ejection fraction, IQR—interquartile range. Values are reported as numbers, values in brackets are percentages, except for LVEF, where a mean value and interquartile range is given, as marked in the appropriate cells. *—statistical significance could not be determined due to the lack of source data and data distribution from the ESSS-SICDI population.

**Table 3:** Comparison of indications for a subcutaneous cardioverter-defibrillator between the study group and the ESSS-SICDI group—Jędrzejczyk–Patej et al. [9].

Indication	Europe	Poland	*p*
Total number of patients; *n* (%)	68 (100)	137 (100)	-
Young age; *n* (%)	34 (50)	104 (75.9)	0.0002
Previous LR complications; *n* (%)	4 (5.9)	12 (8.8)	0.4697
Previous device infection with removal; *n* (%)	1 (1.5)	15 (11)	0.0172
Elevated infection risk; *n* (%)	7 (10.3)	15 (11)	0.8866
Anticipated LR TV-ICD complications; *n* (%)	18 (26.5)	0 (0)	<0.0001
Preservation of vasc. system for future; *n* (%)	3 (4.4)	6 (4.4)	0.9915
No adequate venous access; *n* (%)	0 (0)	10 (7.3)	0.0224
Patient preference; *n* (%)	7 (10.3)	0 (0)	0.0001
Cosmetic advantage; *n* (%)	1 (1.5)	0 (0)	0.1548
Active lifestyle; *n* (%)	7 (10.3)	0 (0)	0.0001
Obesity; *n* (%)	0 (0)	0 (0)	-

LR—lead-related; TV-ICD—transvenous implantable cardioverter-defibrillator. Values are reported as numbers, values in brackets are percentages.

## Data Availability

Data are available on request from the corresponding author.
